# Elevated rate of suicide risk in individuals with opioid use disorder

**DOI:** 10.1111/ajad.70027

**Published:** 2025-03-11

**Authors:** Max Spaderna, Elana Rosenthal, Sun Jung Kang, Rahwa Eyasu, Emade Ebah, Onyinyechi Ogbumbadiugha, Phyllis Bijole, Amelia Cover, Ashley Davis, Meredith Zoltick, Sita Kottilil, Julia Mount, Catherine Gannon, Jasmine Stevens, Grace Garrett, Meghan Derenoncourt, Tina Liu, Lisa Horowitz, Maryland Pao, Sarah Kattakuzhy

**Affiliations:** ^1^ Department of Psychiatry University of Maryland School of Medicine Baltimore Maryland USA; ^2^ Division of Clinical Care and Research, Institute of Human Virology University of Maryland School of Medicine Baltimore Maryland USA; ^3^ DC Partnership for HIV/AIDS Progress Clinical Research Program Washington District of Columbia USA; ^4^ Genetic Epidemiology Research Branch, Intramural Research Program National Institute of Mental Health Bethesda Maryland USA; ^5^ Washington District of Columbia USA; ^6^ Critical Care Medicine Department, Clinical Center National Institutes of Health Bethesda Maryland USA; ^7^ Office of the Clinical Director, National Institute of Mental Health National Institutes of Health Bethesda Maryland USA

## Abstract

**Background and Objectives:**

Few studies have longitudinally investigated suicide risk (SR) in opioid use disorder (OUD). This investigation administered three screening tools to individuals with OUD to compare rates of and variables associated with SR over 12 months.

**Methods:**

121 individuals meeting criteria for OUD within the past 3 years were administered Item #9 of Patient Health Questionnaire‐9 (PHQ‐Item‐9), the twelfth item of DSM‐5‐TR Self‐Rated Level 1 Cross‐Cutting Measure (CCSM‐Item‐12), and the Ask Suicide‐Screening Questions (ASQ) to detect SR at Day 0 and Months 6 and 12. A partitioned generalized methods of moment (GMM) model identified variables associated with SR.

**Results:**

At Day 0, screen‐positive rates for SR were 30% for ASQ, 12.4% for PHQ‐Item‐9, and 4.1% for CCSM‐Item‐12. Rates were similar at Months 6 and 12. Variables significantly associated with SR by PHQ‐Item‐9 were intentional overdose history (*p* < .001), poor sleep (*p* < .001), meeting criteria for psychosis (*p* < .001), and meeting criteria for mania (*p* = .005). Variables significantly associated with SR by ASQ were intentional overdose history (*p* < .001), female gender (*p* = .003), meeting criteria for psychosis (*p* = .001), and total PHQ‐9 score (*p* = .032). Too few participants endorsed SR by CCSM‐Item‐12 to be included.

**Discussion and Conclusions:**

In the OUD population, screening positive for SR was unchanged over 1 year, but detection rates varied by screening tool. History of intentional opioid overdose is independently associated with screening positive for SR.

**Scientific Significance:**

This is the first study to evaluate SR in the OUD population using more than one screening tool, and to show an association of history of intentional opioid overdose with SR.

## INTRODUCTION

Suicide is a global public health problem and a leading cause of death.^1^ Studies have shown that suicidal thoughts and behavior are significant risk factors for suicide risk (SR),^2^ and important for the clinical pathway for suicide detection because these are the only symptoms that can be detected before suicide completion. Individuals with opioid use disorder (OUD) have a higher rate of SR than those without OUD.^3^ Implementing clinical pathways with validated screening instruments for detecting SR is crucially needed to inform interventions for preventing suicide in the OUD population. There are several evidence‐based screening tools commonly used for identifying individuals with SR.

The Patient Health Questionnaire‐9 (PHQ‐9)^4^ is a nine‐item depression‐screening instrument adapted from the Diagnostic and Statistical Manual (DSM) criteria for depressive disorders that has been validated for detecting depression severity.^5^ The ninth item of PHQ‐9 (PHQ‐Item‐9) asks the participant how often they have been bothered by “Thoughts that you would be better off dead or of hurting yourself in some way.” This ninth item has been used to detect SR in patients with substance use disorders with inconsistent results.^6^ Although commonly used as a way to screen for SR, PHQ‐Item 9 has limitations because of how the question is worded.^7^

The DSM‐5‐TR Self‐Rated Level 1 Cross‐Cutting Measure (CCSM)^8^ is a self‐report questionnaire of 13 psychiatric domains. Like PHQ‐9, it has a single item, the twelfth item (CCSM‐Item‐12), that asks the participant how bothered they have been by “Thoughts of actually hurting yourself” over the past 2 weeks. To date, there are scant data on utilizing CCSM to detect suicidal thoughts,^9^ and no study has used CCSM‐Item‐12 to detect SR in the OUD population.

The Ask Suicide‐Screening Questions (ASQ)^10^ is a validated, four‐item self‐report questionnaire designed specifically to detect SR.^11^ ASQ's first three questions inquire about suicidal thoughts, and its fourth asks about previous suicide attempts. An affirmative response to any of the four questions is considered a positive screen for SR, prompting a fifth acuity item asking the participant if they are having thoughts of killing their self “right now.” A yes to the 5th question is considered an “acute” positive, whereas a “no” is considered a “non‐acute” positive. The validity and feasibility of ASQ have been demonstrated in adult inpatient^12^, ^13^, ^14^ and outpatient settings,^15^ as well as in a substance use population,^16^ but ASQ has not been studied specifically in the OUD population.

The primary aims of this analysis are to compare rates of screening positive for SR as detected by PHQ‐Item‐9, CCSM‐Item‐12, and ASQ over a 1‐year period in a prospective cohort of individuals with OUD evaluated in outpatient settings, and to identify variables associated with screening positive for SR by each of these three screening tools.

## METHODS

### Trial design

Long‐term Outcomes of Opioid‐using Persons (LOOP) is a 2‐year prospective, natural‐history cohort study designed to characterize a community sample of individuals with OUD. Participants were recruited from three locations: an urban syringe‐service and buprenorphine‐treatment program, an urban opioid‐treatment program, and a rural, mobile buprenorphine‐treatment program. Participants were eligible for LOOP if they were at least 18 years old and met diagnostic criteria for OUD within the past 3 years. They were excluded if they could not comply with research study visits or had a condition the investigators determined would prevent them from participating in the study. All participants provided informed consent and were seen every 6 months for a 2‐year period. LOOP was approved by the University of Maryland Institutional Review Board. While the study enrolled 200 participants, this sub‐analysis is limited to those who had complete data from the Day 0, Month 6, and Month 12 timepoints.

### Assessments/Screeners

#### Demographics

At baseline, participants provided demographic information, including gender, race, sexual orientation, highest level of education, housing status, and sources of income. Variables were assessed at Day 0, Month 6, and Month 12.

#### Trauma

Current physical abuse was assessed by asking the participant “how often does anyone, including family, physically hurt you?” Physical and sexual trauma before the age of 17 was assessed with the Childhood Trauma Questionnaire^17^ at a single timepoint. This was administered as an opt‐out survey due to the sensitive nature.

#### Suicide risk

Screening positive for SR was defined as an affirmative answer either to the PHQ‐Item‐9, CCSM‐Item‐12, or any question of the ASQ. These screening tools were administered at Day 0, Month 6, and Month 12. A participant was considered an acute positive screen if they answered “yes” to ASQ Item #5, “Are you having thoughts about killing yourself right now.” Participants identified as having SR were immediately evaluated by a clinician with appropriate clinical interventions as necessary.

#### Substance use

Participants were asked how often they use heroin or non‐prescribed opioids, if they were prescribed medications for OUD, and if they had ever intentionally overdosed from heroin or other opioids. OUD severity was assessed with scores using OUD criteria from the DSM‐5.^18^ OUD scores range from 0 to 11, with higher scores indicating greater OUD severity. Hazardous alcohol use was assessed with the Alcohol Use Disorders Identification Test, a 3‐item screening questionnaire for alcohol consumption validated for identifying hazardous alcohol use.^19^ A urine drug screen (UDS) was obtained to detect the presence of non‐prescribed opioids including fentanyl, cocaine, amphetamines, barbiturates, benzodiazepines, and phencyclidine (PCP). Substance use outcomes were assessed at Day 0, Month 6, and Month 12, except for intentional overdose history, which was only assessed at Day 0.

#### Comorbid mental health

Participants were asked if they were receiving psychiatric medications at Day 0, Month 6, and Month 12. Depression symptoms were assessed using the summed score from the nine items of the PHQ‐9.^18^ Mania and psychosis were assessed using the CCSM.^8^ Psychiatric symptoms were assessed at Day 0, Month 6, and Month 12.

#### Health perception and sleep quality

Health perception was assessed with the 36‐Item Medical Outcome Study Short Form Survey (SF‐36),^20^ a 36‐item self‐report questionnaire for quality of life that has been used in various OUD populations.^21^, ^22^, ^23^, ^24^ The SF‐36 General Health score ranges from 0 to 100, with higher scores denoting better general health perception. In the medical literature, the SF‐36 General Health score in the OUD population ranges from 39.2 to 55.1.^21^, ^22^, ^23^ Sleep quality was assessed with the Pittsburgh Sleep Quality Index (PSQI), a validated self‐report questionnaire assessing sleep habits for the majority of days and nights during the past month.^25^ Health perception and sleep quality were assessed at Day 0, Month 6, and Month 12.

### Statistical analysis

The frequencies of variables were calculated at each timepoint, and differences between Day 0 and either Month 6 or Month 12 were determined using χ^2^ tests or Fisher exact tests when appropriate. Associations for the outcomes of interest—a positive screen on PHQ‐Item‐9, CCSM‐Item‐12, and ASQ—were estimated using a partitioned generalized methods of moments (GMM) model.^26^ The GMM model determines how fixed and time‐dependent variables affect the dependent variable. For time‐dependent variables, the model determines how these affect the dependent variable at the current time‐point (in this study, how the variable affects SR at Day 0, Month 6, and Month 12), at the one‐time‐point lag (how the variable at Day 0 affects SR at Month 6, or how the variable at Month 6 affects SR at Month 12), and at the two‐time‐point lag (how the variable at Day 0 affects SR at Month 12). Variables included in this model were gender, housing status, total PHQ‐9 score, meets criteria for psychosis by CCSM, meets criteria for mania by CCSM, SF‐36 General Health score, meets criteria for poor sleeper by PSQI, OUD score, frequency of opioid use, UDS positive for cocaine, and intentional overdose history. Total PHQ‐9 score was not included in the GMM model for PHQ Item‐9 because the procedure failed to find the maximum likelihood of the model, meaning the model did not converge. Variables were selected based on univariate analysis in combination with biologic plausibility. All analyses were performed using SAS software (version 9.4).

## RESULTS

### Demographics

Demographics for the 121 participants that met criteria and were included in this analysis are shown in Table 1. The median age was 57 (IQR = 11.5, range 21–70); 64.5% were male, and 81.0% were Black. Two‐thirds (65.3%) completed at least a high school level of education, 44.6% were unstably housed, and 45.5% received some form of income from the government including unemployment, welfare, and pension benefits. Over one‐third (38.0%) reported a history of sexual and/or physical abuse before age 17, and 15.7% reported current physical abuse. There were no differences in demographics at Months 6 and 12 compared with Day 0.

**Table 1:** Demographics.

Characteristic	No. (%)	No. (%) at Month 6	Change from Day 0 to Month 6 *p‐*value	No. (%) at Month 12	Change from Day 0 to Month 12 *p‐*value
Total	121	N/A		N/A	
Age, median (IQR)	57 (11.5)				
Gender					
Female	40 (33.1)	N/A		N/A	
Male	78 (64.5)				
Other	3 (2.5)				
Race					
Black	98 (81.0)	N/A		N/A	
White	13 (10.7)				
More than one race/other	10 (8.3)				
Sexual orientation					
Heterosexual	111 (91.7)	N/A		N/A	
Not heterosexual	10 (8.3)				
Highest level of education					
Less than high school	42 (34.7)	N/A		N/A	
High school or more	79 (65.3)				
Housing status					
Stable	67 (55.4)	73 (60.3)	0.610	70 (57.9)	0.697
Unstable	54 (44.6)	48 (39.7)		51 (42.2)	
Sources of income					
No income	18 (14.9)	17 (14.1)	0.159	10 (8.3)	0.111
Employment	7 (5.8)	11 (9.1)		14 (11.6)	
Government: unemployment, welfare, pensions	55 (45.5)	68 (56.2)		67 (55.4)	
Other: illegal, mate, family, friends	13 (10.7)	6 (5.0)		10 (8.3)	
Mixed: employment, government, other	28 (23.1)	19 (15.7)		20 (16.5)	
Physically abused by someone					
Never	102 (84.3)	107 (88.4)	0.121	107 (88.4)	0.512
Rarely	9 (7.4)	5 (4.1)		9 (7.4)	
Sometimes	2 (1.7)	7 (5.8)		2 (1.7)	
Fairly often	4 (3.3)	2 (1.7)		3 (2.5)	
Frequently	2 (1.7)	0 (0)		0 (0)	
Missing	2 (1.7)	0 (0)		0 (0)	
Sexual and/or physical abuse before age 17					
Yes	46 (38.0)	N/A		N/A	
No	67 (55.4)				
Missing	8 (6.6)				
Mean OUD score (Std Dev)	4.4 (3.7)	3.1 (3.6)	**0.006**	3.0 (3.5)	**0.003**
Frequency of opioid use in the past month					
None in the past month	54 (44.6)	57 (47.1)	0.868	54 (44.6)	0.196
Once a week or less	29 (24.0)	29 (24.0)		39 (32.2)	
More than once a week, less than once a day	17 (14.1)	13 (10.7)		7 (5.8)	
Once a day	9 (7.4)	9 (7.4)		5 (4.1)	
2–3 times a day	9 (7.4)	7 (5.8)		12 (9.9)	
More than 3 times a day	3 (2.5)	6 (5.0)		4 (3.3)	
Prescribed medications for OUD					
Yes	102 (90.1)	105 (86.8)	0.524	112 (92.6)	0.407
No	12 (9.9)	16 (13.2)		9 (7.4)	
Urine drug screen positive for opioids					
Yes	77 (63.6)	75 (62.0)	0.712	76 (62.8)	0.619
No	38 (31.4)	41 (33.9)		43 (35.5)	
Missing	6 (5.0)	5 (4.1)		2 (1.65)	
Urine drug screen positive for cocaine					
Yes	57 (47.1)	56 (46.3)	0.845	65 (53.7)	0.439
No	58 (47.9)	60 (49.6)		54 (44.6)	
Missing	6 (5.0)	5 (4.1)		2 (1.7)	
Urine drug screen positive for other drugs^a^					
Yes	20 (16.5)	16 (13.2)	0.434	18 (14.9)	0.618
No	94 (77.7)	100 (82.6)		101 (83.5)	
Missing	7 (5.8)	5 (4.1)		2 (1.7)	
Hazardous alcohol use					
Yes	38 (31.4)	29 (24.0)	0.196	23 (19.0)	0.026
No	83 (68.6)	92 (76.0)		98 (81.0)	
Intentional overdose history					
Yes	10 (8.3)		N/A		N/A
No	108 (89.3)				
Missing	3 (2.5)				
Taking psychiatric medications					
Yes	46 (38.0)	54 (55.0)	0.271	49 (59.5)	0.693
No	75 (62.0)	66 (45.0)		72 (40.5)	
Mean PHQ‐9 Score (Std Dev)	7.5 (6.8)	6.9 (6.9)	0.496	7.1 (6.9)	0.650
Meets criteria for psychosis by CCSM					
Yes	19 (15.7)	20 (16.5)	0.861	20 (16.5)	0.861
No	102 (84.3)	101 (83.5)		101 (83.5)	
Meets criteria for mania by CCSM					
Yes	56 (46.3)	43 (35.5)	0.089	39 (32.2)	**0.025**
No	65 (53.7)	78 (64.5)		82 (67.8)	
Mean SF‐36 general health score (Std Dev)	57.7 (21.1)	57.9 (22.5)^b^	0.943	58.2 (24.7)	0.866
Meets criteria for poor sleeper by PSQI					
Yes	77 (63.6)	74 (61.2)	0.691	81 (66.9)	0.589
No	44 (36.4)	47 (38.8)		40 (33.1)	

*Note*: Statistical significance is defined by *p* < .05. Values in bold indicate statistically significant results.

Abbreviations: CCSM, DSM‐5‐TR Self‐Rated Level 1 Cross‐Cutting Measure; IQR, interquartile range; N/A, not available; PHQ‐9, Patient Health Questionnaire‐9; PSQI, Pittsburgh Sleep Quality Index; SF‐36, 36‐Item Medical Outcome Study Short Form Survey; Std Dev, standard deviation.

^a^ Includes amphetamines, barbiturates, benzodiazepines, and phencyclidine.

^b^ Contains 120 observations.

#### Substance use

At Day 0, the mean OUD score was 4.4 (Std Dev = 3.7), consistent with moderate‐severity OUD. Over half (55.4%) of participants reported using opioids at least monthly, including 17.4% who reported using opioids at least daily. Almost all (90.1%) participants were prescribed medications for OUD. UDS at Day 0 was positive for opioids in the majority of participants (63.6%), with cocaine‐positive in 47.1% and other drugs in 16.5% of participants. Nearly a third (31.4%) of participants met criteria for hazardous alcohol use, and 8.3% reported an intentional overdose history. Compared with Day 0, the mean OUD score was lower at Month 6 (3.1; *p* = .006) and Month 12 (3.0; *p* = .003), and fewer participants met criteria for hazardous alcohol use at Month 12 (23; *p* = .026). There were no other differences in substance use outcomes at Months 6 and 12 (Table 1).

#### Mental health, health perception, and sleep quality

At Day 0, 38.0% of participants were taking psychiatric medications. The mean total PHQ‐9 score was 7.5 (Std Dev = 6.8). According to CCSM, 15.7% met criteria for psychosis, and 46.3% met criteria for mania. The mean SF‐36 General Health score was 57.7 (Std Dev = 21.1), which was slightly higher than the values reported in the medical literature for the OUD population.^21^, ^22^, ^23^ Almost two‐thirds (63.6%) met criteria for poor sleep by PSQI. Compared with Day 0, there were no differences in the SF‐36 General Health score or poor sleep quality by PSQI at Months 6 and 12, but fewer participants met criteria for mania by CCSM at Month 12 (39; *p* = .025) (Table 1).

### Detection of suicide risk

#### Day 0

**Figure 1:**
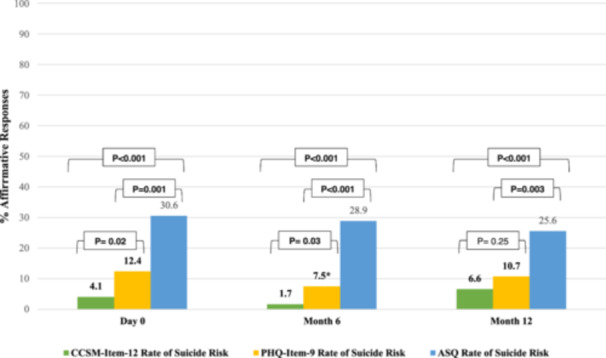
Rates of suicide risk by PHQ‐Item‐9, CCSM‐Item‐12, and ASQ at Day 0, Month 6, and Month 12. Statistical significance is defined by *p* < .05.

The rate of screening positive for SR was 12.4% for PHQ‐Item‐9, 4.1% for CCSM‐Item‐12, and 30.6% for ASQ. The rate was higher for ASQ than for CCSM‐Item‐12 (*p* < .001) and PHQ‐Item‐9 (*p* = .001). It was higher for PHQ‐Item‐9 than for CCSM‐Item‐12 (*p* = .020) (Figure 1).

#### Month 6

120 participants gave responses for PHQ‐Item‐9, and 121 participants gave responses to ASQ and CCSM‐Item‐12. The rate of SR was 7.5% for PHQ‐Item‐9, 1.7% for CCSM‐Item‐12, and 28.9% for ASQ. The rate was higher for ASQ than for CCSM‐Item‐12 (*p* < .001) and PHQ‐Item‐9 (*p* < .001). It was higher for PHQ‐Item‐9 than for CCSM‐Item‐12 (*p* = .030) (Figure 1). The rates at Day 0 and Month 6 were similar for PHQ‐Item‐9 (*p* = .204), CCSM‐Item‐12 (*p* = .446), and ASQ (*p* = .779) (Figure 1).

#### Month 12

The rate of SR was 10.7% for PHQ‐Item‐9, 6.6% for CCSM‐Item‐12, and 25.6% for ASQ. The rate was higher for ASQ than for CCSM‐Item‐12 (*p* < .001) and PHQ‐Item‐9 (*p* = .003). It was similar for PHQ‐Item‐9 and CCSM‐Item‐12 (*p* = .254) (Figure 1). The rates at Day 0 and Month 12 were similar for PHQ‐Item‐9 (*p* = .688), CCSM‐Item‐12 (*p* = .392), and ASQ (*p* = .391) (Figure 1).

### Variables associated with suicide risk according to GMM model

#### Variables collected only at Day 0

Intentional overdose history was significantly associated with SR by PHQ‐Item‐9 (*p* < .001). Intentional overdose history (*p* < .001) and female gender (*p* = .003) were significantly associated with SR by ASQ. The number of participants endorsing SR by CCSM‐Item‐12 was too small to be included in the GMM model (Table 2).

**Table 2:** Significant variables associated with suicide risk by each screening tool according to generalized methods of moment model.

Significant variables collected only at Day 0			
**PHQ‐Item‐9**		**Odds ratio (** ***p‐*** **value)**	
Intentional overdose history		66.52; CI: 21.28, 207.94 (<0.001)	
**CCSM‐Item‐12**		**Odds ratio (*p*‐value)**	
None		N/A	
**ASQ**		**Odds ratio (*p*‐value)**	
Intentional Overdose History		6.23; CI: 2.30, 16.84 (<0.001)	
Female gender		90.45; CI: 4.60, 1779.53 (0.003)	

**Significant time‐dependent variables**			
**PHQ‐Item‐9**	**Current timepoint odds ratio (*p*‐value)**	**1‐Timepoint‐lag odds ratio (*p*‐value)**	**2‐Timepoint‐lag odds ratio (*p*‐value)**
Poor sleeper by PSQI	18.81; CI: 4.67, 75.69 (**<0.001**)	0.02; CI: 0.01, 0.07 (**<0.001**)	3.59; CI: 2.00, 6.43 (**<0.001**)
Meets criteria for psychosis by CCSM	23.12; CI: 10.82, 49.37 (**<0.001**)	5.02; CI: 2.15, 11.74 (**<0.001**)	NS
Meets criteria for mania by CCSM	2.37; CI: 1.30, 4.31 (**0.005**)	NS	14.78; CI: 3.03, 72.18 (**<0.001**)
Frequency of opioid use in past month	NS	1.30; CI: 1.04, 1,63 (0.022)	NS
**CCSM‐Item‐12**	**Current time‐point (*p*‐value)**	**1‐Timepoint‐lag odds ratio (*p*‐value)**	**2‐Timepoint‐lag odds ratio (*p*‐value)**
None	N/A	N/A	N/A
**ASQ**	**Current time‐point (*p*‐value)**	**1‐Timepoint‐lag odds ratio (*p*‐value)**	**2‐Timepoint‐lag odds ratio (*p*‐value)**
Meets criteria for psychosis by CCSM	5.11; CI: 1.92, 13.57 (**0.001**)	NS	NS
Total PHQ‐9 score	1.05; CI: 1.00, 1.10 (**0.032**)	NS	NS

*Note*: Statistical significance is defined by *p* < .05. Values in bold indicate statistically significant results.

Abbreviations: ASQ, Ask‐Suicide‐Screening Questions; CCSM‐Item‐12, Twelfth Item of DSM‐5‐TR Self‐Rated Level 1 Cross‐Cutting Measure; CCSM, DSM‐5‐TR Self‐Rated Level 1 Cross‐Cutting Measure; CI, confidence interval; N/A, not available (n was too small to be included in the GMM model); NS, not statistically significant (p‐value is >.05); PHQ‐Item‐9, Ninth Item of Patient Health Questionnaire‐9; PHQ‐9, Patient Health Questionnaire‐9; PSQI, Pittsburgh Sleep Quality Index.

#### Variables at current timepoint

Poor sleeper by PSQI (*p* < .001), meeting criteria for psychosis by CCSM (*p* < .001), and meeting criteria for mania by CCSM (*p* = .005) were significantly associated with SR by PHQ‐Item‐9. Meeting criteria for psychosis by CCSM (*p* = .001) and total PHQ‐9 score (*p* = .032) were significantly associated with SR by ASQ (Table 2).

#### Variables at 1‐timepoint lag

Poor sleeper by PSQI (*p* < .001), meeting criteria for psychosis by CCSM (*p* < .001), and frequency of opioid use in the past month (*p* = .022) were significantly associated with SR by PHQ‐Item‐9. None of the variables were significantly associated with SR by ASQ.

#### Variables at 2‐timepoint lag

Poor sleeper by PSQI (*p* < .001) and meeting criteria for mania by CCSM (*p* < .001) were significantly associated with SR by PHQ‐Item‐9. None of the variables were significantly associated with SR risk by ASQ.

### ASQ items

No one answered “yes” to ASQ Item #5, “Are you having thoughts about killing yourself right now,” at any timepoint, so none of the participants screened acute positive. Of the people who screened positive for ASQ, 23.1% at Day 0, 20.7% at Month 6, and 19.8% at Month 12 answered “yes” to ASQ Item #4, “Have you ever tried to kill yourself?” Females were more likely than males to answer “yes” to ASQ Item #4 at Day 0 (37.5% vs. 19.2%; *p* = .031), Month 6 (35% vs. 14.1%; *p* = .009), and Month 12 (32.5% vs. 14.1%; *p* = .019). At Day 0, 90% of those with an intentional overdose history answered “yes” to ASQ Item #4, but the proportion of those with an intentional overdose history answering “yes” to Item #4 declined to 70% at Months 6 and 12.

## DISCUSSION

In this cohort of older individuals with mild to moderate‐severity OUD evaluated over 1 year, we found significant differences in the rate of SR by each screening tool, with ASQ detecting the highest rate followed by PHQ‐Item‐9 and CCSM‐Item‐12. Though ASQ detected the highest rate of SR risk, none of the participants had acute or active suicidal ideation at any timepoint. Using results from the screening tools, we identified several risk factors associated with SR. Importantly, an intentional overdose history was associated with screening positive for SR, underlying the necessity of screening for SR explicitly in the OUD population. Other risk factors associated with SR were female gender, poor sleep, psychosis, mania, depression, and frequency of opioid use.

Previous investigations in prospective cohorts of individuals with OUD have found high rates of SR, but these administered only one screening tool.^27^, ^28^ By administering three screening tools to individuals with OUD over a 1‐year period, we could determine differences in how each screening tool measures SR. Our results show that the rate of SR measured by each screening tool did not significantly change over time, consistent with previous research.^27^, ^28^

ASQ identified far more people with SR than either PHQ‐Item‐9 or CCSM‐Item‐12. This is because ASQ identifies both those who have previously engaged in suicidal behavior with ASQ Item #4, and those currently experiencing passive and active suicidal ideation with the other ASQ items. In contrast, the wording for PHQ‐Item‐9 and CCSM‐Item‐12 asks only about passive thoughts of death and/or non‐suicidal self‐injury. As a result, there is a stark difference between the screening tools, with ASQ identifying 7.5 times more participants with SR than CCSM‐Item‐12 at Day 0. In this analysis, we cannot comment on whether ASQ is better at detecting SR or identifying more false positives, but our results suggest that the wording of CCSM‐Item‐12 and PHQ‐Item‐9 might miss people at risk for SR^67^. More prospective studies following individuals with OUD over extended periods of time are needed to determine the effectiveness and accuracy of screening tools for SR in predicting suicide attempt and completion in the OUD population, and which screening tools should be considered the gold standard.

To our knowledge, this is the only investigation to use ASQ, an instrument designed specifically for detecting SR, in the OUD population. Our results found the rate of SR by ASQ to be double that of hospitalized adult medical patients,^12^ demonstrating the need for effective interventions to mitigate SR in the OUD population. Importantly, none of the participants were identified as having imminent suicidal ideation indicating acute SR, a finding consistent with another study using ASQ to screen individuals with substance use disorders.^16^ While a large percentage of patients with OUD may require referral to mental health services, emergency evaluation, an immediate intervention challenging to implement for some providers, may be only rarely needed.^16^ This indicates that screening for SR could prioritize individuals with OUD who require psychiatric engagement without adding significant strain to the emergency healthcare system.

Our investigation emphasizes the importance of asking patients about intentional overdose history, which was associated with SR by ASQ and PHQ‐Item 9. Crucially, this association cannot be attributed solely to a self‐report of suicide attempts. Not all the participants endorsing an intentional overdose history reported a history of suicide attempts by ASQ, and the proportion reporting both a history of intentional overdose and suicide attempts declined over the 1‐year study period. One possible explanation for this decline is that people who use substances might not consider an intentional opioid overdose to be a suicide attempt. Studies have shown that individuals with a history of intentional opioid overdose report varying levels of suicidal ideation before an overdose.^29^ Therefore, providers should ask patients with OUD about their intentional overdose history, as this may provide important information that could inform their SR beyond the questions currently included in the screening tools.

Our cohort had high rates of psychiatric illness and poor sleep, though the 46.3% screening positive for mania is likely inaccurate and reflects an overdiagnosis of bipolar disorder commonly observed in substance use disorders.^30^ Nevertheless, psychiatric symptoms, including depression, mania, poor sleep, and psychosis, were associated with SR. This aligns with previous research showing that psychiatric illness and poor sleep increase SR in the OUD population.^27^, ^31^, ^32^ Despite this increased risk, individuals with OUD have not received the necessary and recommended mental health services. For instance, in the US individuals with suicidal ideation were 2.5 times more likely not to receive mental health treatment than those without suicidal ideation.^33^ This highlights the importance of identifying individuals with OUD and SR, and connecting them to psychiatric treatment.

Conversely, substance use outcomes were weakly associated with SR in our study. While opioid use may explain differences in SR between individuals who do and do not use opioids illicitly,^34^ frequency and severity of use do not fully account for the disparity in risk among patients with opioid use. As such, our study suggests that interventions to improve access to and delivery of psychiatric care may help mitigate SR among people with OUD. Moreover, while harm reduction efforts, such as the distribution of naloxone and fentanyl test strips, are critical for preventing overdose‐related deaths, the one‐in‐ten patients acknowledging a history of intentional overdose in this study suggest that interventions addressing mental health may help curb the precipitous rise in overdose fatalities.

Our study revealed a gender divide, as the female gender was strongly associated with SR by ASQ and not PHQ‐Item‐9. Because ASQ asks about a history of suicide attempts, it makes sense that ASQ would identify more females with SR. Females in our study were twice as likely to endorse a history of suicide attempt as males, a finding consistent with previous research showing that females are at least one‐and‐a‐half times more likely to attempt suicide than males.^35^ More investigation is needed into the gender differences of screening tools for SR and whether these differences should be considered when implementing screening tools for SR in an OUD population.

There are several limitations to our study. Patients were predominantly Black, older, and living in urban environments, so these findings may not be generalizable to other subsets of the OUD population. Data were collected at three timepoints over a 12‐month period, so it is unclear how SR might change beyond this timeframe. Study participants were asked about their intentional overdose history only when they entered the LOOP investigation. This may have influenced their self‐report of suicide attempts at that timepoint because of recall bias. The screening tools in this study should not be conflated with structured assessments, which more thoroughly evaluate the presence of SR. Finally, the responses in this investigation were collected by self‐report, not validated with other objective data, and subject to bias.

In conclusion, data from the LOOP investigation demonstrate that people with OUD should be screened for SR. Our study shows that the rates of SR identified by each screening tool did not significantly change over a 1‐year period, underscoring a chronicity of SR in people with OUD. There is significant variation in how screening tools identify SR in the OUD population. ASQ, which asks about both suicidal thoughts and previous suicide attempts, identified far more participants than either PHQ‐9‐Item 9 or CCSM‐Item‐12, which only asks about suicidal thoughts. Despite having the highest rate of SR, ASQ did not identify anyone as an acute SR, suggesting screening tools for SR can be administered to individuals with OUD without the need for emergent intervention. Our results emphasize the importance of identifying SR by asking individuals with OUD specifically about a history of intentional overdose, and of identifying psychiatric illness in individuals with OUD and referring them to treatment. Future research is needed to determine the specific impact of screening positive for SR and if screening individuals with OUD for SR can decrease their likelihood of future suicide attempts.

## CONFLICT OF INTEREST STATEMENT

The authors declare no conflicts of interest. The authors alone are responsible for the content and writing of this paper.
